# Enhanced Acylcarnitine Annotation in High-Resolution Mass Spectrometry Data: Fragmentation Analysis for the Classification and Annotation of Acylcarnitines

**DOI:** 10.3389/fbioe.2015.00026

**Published:** 2015-03-09

**Authors:** Justin J. J. van der Hooft, Lars Ridder, Michael P. Barrett, Karl E. V. Burgess

**Affiliations:** ^1^Glasgow Polyomics, University of Glasgow, Glasgow, UK; ^2^Laboratory of Biochemistry, Wageningen University and Research Centre, Wageningen, Netherlands

**Keywords:** high-resolution mass spectrometry, collisional fragmentation, metabolite annotation, classification, acylcarnitines, human urine, HILIC chromatography, metabolomics

## Abstract

Metabolite annotation and identification are primary challenges in untargeted metabolomics experiments. Rigorous workflows for reliable annotation of mass features with chemical structures or compound classes are needed to enhance the power of untargeted mass spectrometry. High-resolution mass spectrometry considerably improves the confidence in assigning elemental formulas to mass features in comparison to nominal mass spectrometry, and embedding of fragmentation methods enables more reliable metabolite annotations and facilitates metabolite classification. However, the analysis of mass fragmentation spectra can be a time-consuming step and requires expert knowledge. This study demonstrates how characteristic fragmentations, specific to compound classes, can be used to systematically analyze their presence in complex biological extracts like urine that have undergone untargeted mass spectrometry combined with data dependent or targeted fragmentation. Human urine extracts were analyzed using normal phase liquid chromatography (hydrophilic interaction chromatography) coupled to an Ion Trap-Orbitrap hybrid instrument. Subsequently, mass chromatograms and collision-induced dissociation and higher-energy collisional dissociation (HCD) fragments were annotated using the freely available MAGMa software^1^. Acylcarnitines play a central role in energy metabolism by transporting fatty acids into the mitochondrial matrix. By filtering on a combination of a mass fragment and neutral loss designed based on the MAGMa fragment annotations, we were able to classify and annotate 50 acylcarnitines in human urine extracts, based on high-resolution mass spectrometry HCD fragmentation spectra at different energies for all of them. Of these annotated acylcarnitines, 31 are not described in HMDB yet and for only 4 annotated acylcarnitines the fragmentation spectra could be matched to reference spectra. Therefore, we conclude that the use of mass fragmentation filters within the context of untargeted metabolomics experiments is a valuable tool to enhance the annotation of small metabolites.

## Introduction

Mass spectrometry in conjunction with liquid chromatography has been successfully used for two decades to profile extracts of complex biological samples. In recent years, the ability to identify and annotate hundreds of compounds simultaneously in a single sample has been a major driving force behind the expansion of the technology known as metabolomics (Dunn et al., 2013). However, assigning molecular structures to detected mass signals has proven to be a primary challenge in metabolomics studies (Van Der Hooft et al., 2013). Modern mass spectrometers are capable of capturing the molecular masses of ionized metabolites at high-resolution, providing scientists with an unprecedented insight in complex biological mixtures such as cell extracts, plasma, or urine. High-resolution mass spectrometers such as the orbitrap provide accurate mass measurements and are thus able to reliably distinguish co-eluting isobaric species of marginally different mass (Watson and Sparkman, 2007; Makarov and Scigelova, 2010), in contrast to mass spectrometers with nominal mass detection. Furthermore, many modern mass spectrometers can trap ionized metabolites in collision cells and generate fragments that can be analyzed at high-resolution too (Schuhmann et al., 2011; Van Der Hooft et al., 2011, 2012b). Analysis of the resulting fragments and neutral losses usually provides additional structural information about the fragmented mass as well as more constraints for its elemental formula.

Recent studies have demonstrated the use of an ion trap – orbitrap hybrid mass spectrometer to fragment reference compounds or metabolites present in biological extracts (Kasper et al., 2012; Rojas-Cherto et al., 2012; Van Der Hooft et al., 2012a). Two types of fragmentation are commonly employed in metabolomics studies: collision induced dissociation (CID) or higher-energy collisional dissociation (HCD), each of them usually providing slightly different fragmentation spectra for the same fragmented metabolite. High-resolution mass spectrometry combined with fragmentation greatly enhances our ability to structurally elucidate compounds, but assigning a chemical annotation to observed mass features remains a major bottleneck when using untargeted metabolomics approaches (Kind and Fiehn, 2010; Wishart, 2011; Dunn et al., 2013; Van Der Hooft et al., 2013).

Although untargeted metabolomics studies aspire to capture and characterize the entire metabolome of a biological sample, in practice trade-offs are made during sample preparation and mass detection as to which metabolites are actually measured. Complex urine extracts prepared for “normal phase” chromatography (i.e., hydrophilic interaction chromatography, HILIC) typically contain several thousands of small polar metabolites covering multiple chemical classes that can be separated and detected in a mass chromatogram (Creek et al., 2011; Zhang et al., 2012). Here, we describe the annotation of multiple detected acylcarnitines using global HILIC – high-resolution tandem mass spectrometry approaches. Acylcarnitines are all derivatives of carnitine, carrying different fatty acids (Thompson et al., 2012). These metabolites play an important role in energy metabolism, for example, by transporting acyl moieties into the mitochondria where they undergo beta oxidation, and specific enzymes and transporter proteins exist that translocate acylcarnitines in and out of the cells and blood stream and excessive acylcarnitines are excreted in the urine (Frayn, 2010). Acylcarnitine concentrations vary depending on the energy status of the human body (Thompson et al., 2012). Irregular urinary and serum acylcarnitine patterns have been identified as biomarkers for several energy related diseases including diabetes mellitus (Dudzik et al., 2014) and metabolic syndrome (Patterson et al., 2009; Peng et al., 2013a). Moreover, acylcarnitine patterns were found to be markers for inborn metabolic distortions caused by malfunctioning of enzymes involved in fatty acid metabolism (Ellis et al., 2007; Gucciardi et al., 2012).

Both reversed phase (C18 based) and HILIC chromatography, often combined with dedicated sample preparation, have been used to separate acylcarnitines from other urinary metabolites and from each other (Yang et al., 2007; Gucciardi et al., 2012; Peng et al., 2013b). Gas chromatography coupled to mass spectrometry has also been used successfully to detect and characterize acylcarnitine species (Libert et al., 2000). The most comprehensive study on acylcarnitine species in urine up to date reported over 350 species using a 2 h UPLC run following sample preparation focused specifically on this class of compound, and a targeted nominal mass fragmentation approach (Zuniga and Li, 2011). In contrast, untargeted mass spectrometry experiments aim to identify a diverse spectrum of compounds without being optimized for particular chemical classes. Within this context, the use of collision induced fragmentation of metabolites to assess their structure can serve as a means of annotating substructures or structures to metabolites in complex mixtures. The aim of this study was to explore the use of data dependent and targeted CID and HCD fragmentation in combination with a generic pHILIC or HILIC gradient coupled to untargeted high-resolution mass spectrometry to classify and annotate acylcarnitines present among the broad spectrum of polar metabolites that can be detected in human urine extracts. Furthermore, the generated high-resolution fragmentation spectra were used to obtain structural information on the fatty acyl-moiety linked to the carnitine molecule.

**Figure 1:**
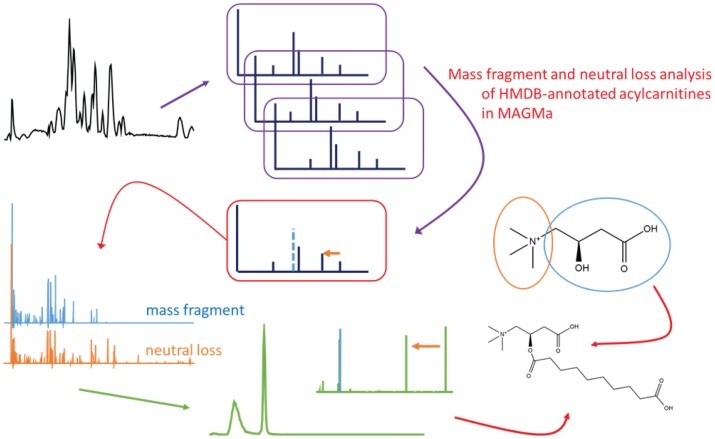
**Graphical representation of the described data analysis approach, starting from top left following the arrows to the bottom right: untargeted mass chromatogram, fragmentation data acquisition, HMDB annotation and fragment annotation in MAGMa to find specific mass fragments and neutral losses for acylcarnitines, mass fragmentation filter and the structure of carnitine, extracted ion chromatogram and neutral loss trace for the acylcarnitine filter, extracted ion chromatogram and spectrum for a detected acylcarnitine, and an example acylcarnitine (conjugate of sebabic acid)**.

Fragmentation spectra obtained from urine extracts were first annotated using the MAGMa software developed by Ridder et al. (2014a) (see text footnote 1) with potential candidates from a compound database containing known and predicted human metabolites (HMDB)^2^. Then, based on fragment annotation as proposed by the MAGMa software and as proposed in literature, key fragments, or losses were determined in order to create a mass fragmentation filter that uniquely screens for the acylcarnitine structures. Subsequently, CID-MS*^n^* and HCD type fragmentation files were manually studied to assess the designed filter for acylcarnitine classification. Next, the mass fragmentation filter was used to annotate acylcarnitines fragmented by data dependent and independent fragmentation. A graphical outline of the approach is presented in Figure 1. All acylcarnitine species that could be annotated in the pHILIC and HILIC gradients are listed in Table S1 in Supplementary Material.

## Materials and Methods

### Materials

HPLC-grade methanol, acetonitrile, isopropanol, and analytical reagent grade chloroform were acquired from Fisher Scientific, Loughborough, UK. HPLC-grade H_2_O was purchased from VWR Chemicals, Fountenay-sous-Bois, France. Formic acid (for mass spectrometry) and ammonium carbonate were acquired from Fluka Analytical (Sigma-Aldrich), Steinheim, Germany. l-carnitine (≥98%) was obtained from Sigma-Aldrich, St. Louis, MO, USA.

Urine samples from anonymized healthy human volunteers were used from a clinical data set in the Glasgow Polyomics archive. The seven urine samples used in this study were numbered 1–7, urines 1–4 were used for data-dependent fragmentation approaches, where urines 5–7 were used for targeted fragmentation of suspected low abundant acylcarnitines, as summarized in Table 1.

**Table 1:** **Schematic experimental design of human urines used in this study**.

Urine #	Chromatography	Mass spectrometry	Usage
1	pHILIC	Untargeted	Filter generation and application
2	pHILIC	Untargeted	Filter generation and application
3	HILIC	Untargeted	Filter application
4	HILIC	Untargeted	Filter application
5	HILIC	Targeted	Filter application
6	HILIC	Targeted	Filter application
7	HILIC	Targeted	Filter application

*These samples were obtained as part of an ethical approved project, granted by the MVLS College Ethics Committee of the University of Glasgow, Glasgow, UK (Project No: 2012061). The volunteers gave their consent for conducting metabolomics studies with their urine samples*.

### Methods

#### l-carnitine solution for direct infusion

From a 100 mM solution in H_2_O, 15 μL was mixed with 5 μL isopropanol in a 96 wells-plate to yield a final 75 mM l-carnitine solution.

#### Urine sample preparations

A general metabolome extraction procedure was performed: (i) 5 μL urine was extracted in 200 μL chloroform/methanol/water (1:3:1) at 4°C; (ii) then vortexed for 5 min at 4°C; (iii) then centrifuged for 3 min (13,000 × *g*) at 4°C. The resulting supernatant was stored at −80°C until analysis.

#### NanoMate direct infusion measurements

To allow for sufficient spray time for extensive fragmentation experiments, a chip-based nano electrospray (Triversa NanoMate, Advion, USA) source was used in infusion mode with a set up as described previously (Van Der Hooft et al., 2011). The key settings were positive ionization mode, sample volume of 10 μL, a gas pressure of 0.5 psi, and a voltage of 1.5 kV, with a data acquisition delay of 0.5 min. Orbitrap Elite FTMS mass spectrometry settings: AGC 1 × 10^6^ (full scan mode) and 5 × 10^4^ (MS*^n^* mode), capillary temperature 220°C, source voltage +1.6 kV, source current 100 μA, S-lens RF 65.5%, skimmer offset 0 V, 1 microscan, and the mass spectrometer was calibrated with Thermo calmix and tuned on *m*/*z* 195.10 (caffeine). Full scan data were acquired for 1 min to check signal intensity and purity of the sample. MS2 fragmentation spectra were obtained for 5 min in positive ionization mode using CID and HCD fragmentation modes from 10 to 200 normalized collision energy (NCE), in steps of 10 NCE. Further key settings were isolation width of 3.0 Da, minimum signal required of 4500, first mass fixed at 50.00 *m*/*z* (HCD), and additional microscans and AGC targets were 2 and 0 (MS2), 4 and 2 × 10^6^ (MS3), and 6 and 3 × 10^6^ (MS4), respectively.

#### HILIC-MS/MS

The LC separation was performed using HILIC (Creek et al., 2011), using the following equipment, gradients, and settings:

(i) ZIC-pHILIC 150 mm × 4.6 mm, 5 μm column (Merck Sequant) equipped with the corresponding pre-column, operated by an UltiMate 3000 RSLCnano liquid chromatography system (Dionex, Camberley, Surrey, UK). The LC mobile phase was a linear biphasic gradient from 80% B to 20% B over 15 min, followed by a 2 min wash with 5% B, and 8 min re-equilibration with 80% B, where solvent B is acetonitrile and solvent A is 20 mM ammonium carbonate in water. The flow rate was 300 μL/min, column temperature was held at 35°C, injection volume was 10 μL, and samples were maintained at 4°C in the autosampler.
(ii) ZIC-HILIC 150 mm × 2.1 mm, 3.5 μm column (Merck Sequant) equipped with the corresponding pre-column, operated by an UltiMate 3000 RSLCnano liquid chromatography system (Dionex, Camberley, Surrey, UK). The LC mobile phase was a biphasic linear gradient from 80% B to 20% B over 30 min, followed by an 8 min wash with 5% B, and 8 min re-equilibration with 80% B, where solvent B is 0.08% formic acid in acetonitrile and solvent A is 0.1% formic acid in water. The flow rate was 100 μL/min, column temperature was held at 35°C, injection volume was 10 μL, and samples were maintained at 4°C in the autosampler.

The Orbitrap Elite mass spectrometer was calibrated using Thermo calibration mix in positive ionization mode and tuned on *m*/*z* 195.10 (caffeine). Source mass spectrometry settings for both ZIC-HILIC and ZIC-pHILIC in positive ionization mode were as follows: a HESI 2 probe was used with AGC 1 × 10^6^ (full scan mode) and 5 × 10^4^ (MS*^n^* mode), sheath gas 10 a.u., auxiliary gas 5 a.u., sweep gas 1 a.u., source heater temperature 150°C, capillary temperature 275°C, source voltage +4 kV, source current 100 μA, S-lens RF 50%, skimmer offset 0 V, maximum ion times of 100 ms (full scan mode) and 200 ms (MS*^n^* mode), and all scans consist of 1 microscan.

Data-dependent ZIC-pHILIC-MS/MS and MS*^n^*: data were obtained in profile mode, for full scans the *m*/*z* window was 70.00–1000.00 and the resolution was set to 240,000. For fragmentation experiments, key settings were: isolation width of 1.0 Da, minimum signal required of 500, first mass fixed at 50.00 *m*/*z* (HCD), and a dynamic exclusion of 48 s. A rejection list was included with the top 15 most intense ions encountered in blank injections preceding the fragmentation runs to reduce the number of non-informative fragmentation spectra. HCD fragmentation spectra of the most intense ion (data-dependent acquisition) in the full scan were obtained at 30, 70, and 110 NCE. CID-MS*^n^* (*n* ≤ 3) fragmentation was performed as in (Van Der Hooft et al., 2012b), but using 45 NCE.

Data-dependent ZIC-HILIC-MS/MS: as for ZIC-pHILIC-MS, with a resolution set to 120,000 for full scan mode, and 15,000 for MS ≥2.

Targeted ZIC-HILIC-MS/MS: as for ZIC-pHILIC-MS/MS, with a parent ion list including masses of potential acylcarnitine structures with retention times comparable to previously annotated acylcarnitine structures in the data-dependent HILIC-MS/MS runs of urine 3 and 4, i.e., eluting between 5 and 7 min. The parent ion lists for urine extracts 5–7 included in total 27 masses not previously fragmented and annotated in urines 1–4 for which the most probable elemental formula matches C_x_H_y_NO_z_, i.e., comprising of one nitrogen atom and no other elements than carbon, hydrogen, and oxygen. The structure of the MS/MS method was as follows: one full scan, followed by fragmentation at 30, 70, and 110 NCE of the two most intense ions present at the parent ion list (i.e., no fragmentation took place if no targeted ions were present above the threshold).

Prior to the hyphenated MS fragmentation experiments, a series of four blanks, quality control samples, and standards mixtures were injected to stabilize the system, determine background ions for the rejection list in data-dependent fragmentation, and check the quality of the chromatographic runs. Accurate masses of standards were obtained well within 5 ppm accuracy.

### Data analysis

Thermo raw data files were checked for the presence of informative fragmentation spectra in Xcalibur version 2.2. Raw data files were then converted into mzXML files (using MM_File_Conversion3)^3^ prior to MAGMa analysis (see text footnote 1) (Ridder et al., 2014b). The MzXML files are available to download in the supplementary information.

#### MAGMa settings and analysis

The MzXML files were uploaded to the MAGMa server and MAGMa default settings were used to annotate urine data-dependent fragmentation files with compounds present in HMDB (updated at April 2014), except for a maximum of 2 allowed water losses (in generated substructures by breaking up to 3 bonds) and minimum intensity threshold levels of 1,500 counts, and 2% of the base peak for substructure annotation. This annotation is based on the detected precursor masses and the detected fragments, and produces proposed fragment annotations with a penalty score that determines the candidate scores and rankings of candidate metabolites (Ridder et al., 2012). CID (MS^*n*^) type files were handled as in Ridder et al. (2014b). Multiple HCD-MS^2^ fragmentation spectra of the same precursor ion, recorded at the different collision energies, were merged by MAGMa as suggested previously (Horai et al., 2010; Wolf et al., 2010). Each analysis took 3 min or less. Annotated acylcarnitines and carnitine-related compounds were extracted from all the MAGMa annotations in the results page by applying a filter on the metabolite names (“carnitine”) in order to count the number of annotated acylcarnitines in each run.

#### Acylcarnitine annotation using the mass fragmentation filter

To scan for compounds that fulfill the defined filter criteria, extracted ion chromatograms and neutral loss traces were created in Xcalibur from the raw data files with a 6 ppm window, to account for less accurate mass values in the lower *m*/*z* range of fragmentation spectra.

#### Metabolite annotation

Metabolites were classified as acylcarnitines if the most likely elemental formula matched the mass fragment and neutral loss filter as described in Section “Acylcarnitine Annotation Using the Mass Fragmentation Filter.” It should be stressed that this study does not intend to fully identify the acylcarnitine molecules, but focuses instead on robust metabolite annotation of acylcarnitines by their classification while obtaining structural information on the acyl moiety [i.e., MSI metabolite identification (MSI MI) level 3 (Sumner et al., 2007; Van Der Hooft et al., 2013)]. MSI MI level 2 can be achieved if the generated fragmentation pattern matched a spectral database spectrum. Full identification would be achievable by obtaining authentic standards or by elaborate concentration and purification from the urine matrix, but falls outside the scope of this study. Scifinder analyses (July 2014) were performed to obtain the number of candidates for (i) the elemental formula, (ii) the elemental formula refined with carnitine as substructure, and (iii) the number of references for the most cited acylcarnitine structure in Scifinder. In addition, for each annotated acylcarnitine, the number of HCD fragments between *m*/*z* 85 and the fragmented precursor mass, the number of oxygen atoms in the acylcarnitine minus the three oxygen atoms in carnitine, and the C:H ratio for each annotated acylcarnitine was determined. Additionally, the presence of matching carnitine metabolites in HMDB was checked (Table S1 in Supplementary Material).

The acyl moiety was annotated by searching for and matching of fragmentation spectra of acylcarnitine reference compounds in the following databases: METLIN^4^, mzCloud^5^, and HMDB^6^. Furthermore, the HMDB-MAGMa annotation was studied, as well as the acyl-derived mass fragments and neutral losses. The resulting metabolite annotations are listed in Table S1 in Supplementary Material.

## Results

We employed a generic metabolite extraction method (Vincent and Barrett, 2015) with untargeted small metabolite pHILIC and HILIC profiling approaches (Creek et al., 2011; Zhang et al., 2012) in combination with HCD-MSMS and CID-MS*^n^* fragmentations to determine whether robust metabolite annotation of small polar metabolites could be established using accurate mass fragmentation spectra. Both pHILIC and HILIC chromatography were included in the study to cover the two routinely used chromatographic separations in our laboratory. As a test case, we have focused on seeking to enhance the annotation of acylcarnitines in the complex mixture human urine. Since acylcarnitines ionize poorly in negative ionization mode, positive ionization mass spectrometry was employed. Two human urine extracts (urine extract 1 and 2) were run using the same pHILIC chromatography, but differing in HCD or CID (MS*^n^*) type fragmentation as specified in the Sections “Methods” and “HILIC-MS/MS.”

### MAGMa annotation with HMDB candidates of untargeted analysis of two urine extracts

From the MzXML files of human urine 1, 615 merged HCD-MS2 and 461 CID-MSn spectra were read by MAGMa (Ridder et al., 2014b), for HCD and CID types of fragmentation, respectively. A total of 413 and 372 candidates from HMDB (Wishart et al., 2013), including both known molecules and structures predicted to be present in human samples, were matched to 292 and 224 precursor ions, respectively. Supported by the substructure-based interpretation of fragment spectra in MAGMa, the annotation of a range of urine metabolites could be confirmed. For example, 4-guanidinobutanoic acid (HMDB03464) and guanidoacetic acid (HMDB00128) were annotated to a fragmented LC-MS peak, both containing a mass fragment that indicates the presence of a guanido group. Those acids are known to be present in human urine. Homocarnosine (HMDB00745) is another MAGMa annotated compound found in urine. Interestingly, by using MAGMa we also annotated acetylcarnosine (HMDB12881), a compound predicted to be present in human samples, but not previously observed or confidently annotated. The dipeptide prolylhydroxyproline (HMDB06695) was annotated based on its precursor peak and mass fragments, including two hydroxyproline fragments ([C_5_H_8_NO_3_]^+^ and [C_4_H_6_N]^+^), providing evidence for the prolylhydroxyproline configuration over the hydroxylprolyl dipeptide. Similarly, four predicted isoleucine/leucine containing dipeptides were annotated, including the isomers alanyl-isoleucine (HMDB28690) and isoleucyl-alanine (HMDB28900) or their leucine analogs. Glycerophosphocholine (HMDB00086) was present as a lower abundance peak in the mass chromatogram, showing distinct fragments for its phosphor-containing fragments. Finally, the annotated l-carnitine (HMDB00062) was one of the major abundant peaks in the chromatogram.

It should be further noted that 323 and 332 HCD precursors, and 237 and 245 CID precursors, for urine 1 and 2, respectively, did not match any candidate from HMDB. Currently, the online HMDB database contains 69 carnitine-related metabolites, of which 36 are described to be present in human samples, the remainder being predicted to be present in humans. The MAGMa metabolite annotation of HMDB compounds to the HCD and CID fragmentation files of urines 1 and 2 resulted in 12 annotated acylcarnitine candidates, of which 3 had one or multiple isomers matched. These candidates included carnitine (HMDB00062) and 3-dehydrocarnitine (HMDB12154). Not all acylcarnitines were annotated in those four fragmentation files due to (i) the stochastic nature of the data-dependent fragmentation, omitting the fragmentation of the mass features annotated with propionylcarnitine (HMDB00824) and tiglylcarnitine (HMDB02366) in the CID fragmentation file of urine 2, and (ii) the biological differences between the two urine files, resulting in different abundance levels for three annotated acylcarnitines. For example, 2-trans,4-cis-decadienoylcarnitine (HMDB13325) was three times more abundant in human urine 1 (1.1E6 vs. 3.3E5 cts), triggering data-dependent fragmentation in human urine 1, but not in human urine 2. We note that CID and HCD fragmentation types were equally informative with respect to HMDB acylcarnitine annotations with MAGMa. Moreover, no other type of structures from HMDB was matched to the annotated acylcarnitine fragmentation spectra.

### MAGMa analysis of pHILIC runs for fragment annotation and construction of a mass fragmentation filter to classify acylcarnitines

**Figure 2:**
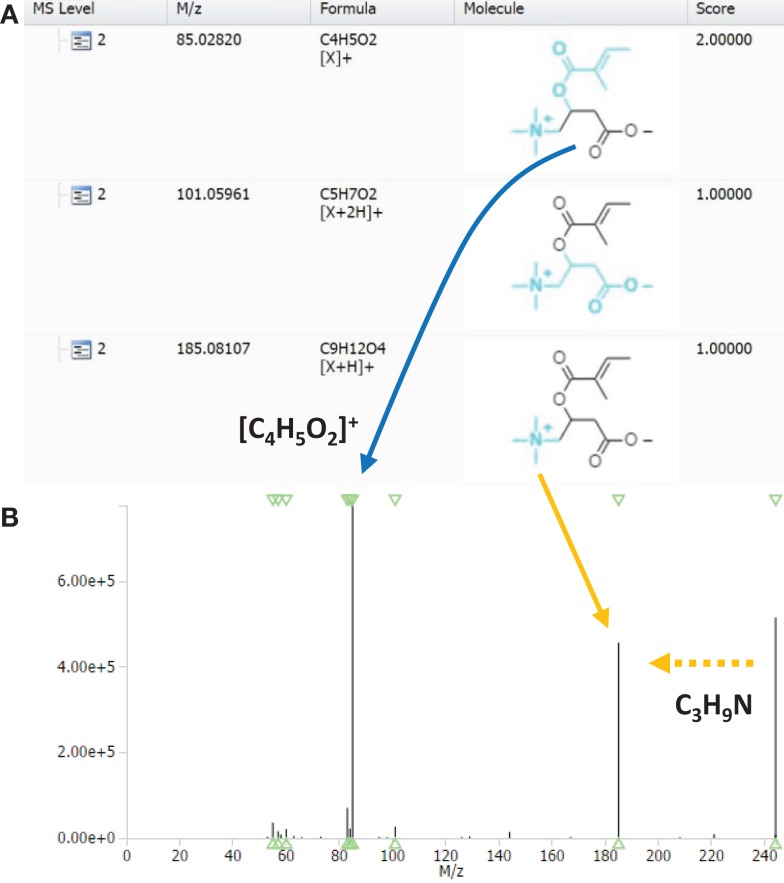
**(A)** Fragment annotation of HMDB-annotated acylcarnitine with elemental formula C_12_H_21_NO_4_ in MAGMa, annotated as tiglylcarnitine (HMDB02366), and **(B)** merged HCD-MS^2^ spectrum of the annotated tiglylcarnitine. Arrows indicate the corresponding mass fragmentation peaks of the neutral loss of trimethylamine (yellow dashed arrow indicates neutral loss in the mass spectrum; the yellow full arrow points to resulting mass fragment) and of the mass fragment C_4_H_5_O_2_ (blue full arrow).

In order to determine key fragments or neutral losses (or a combination thereof) that can be used to screen for acylcarnitines, the fragment annotations as proposed by MAGMa were studied. Figure 2A shows a screenshot of the fragment list of the HMDB-annotated tiglylcarnitine (fragments ≥*m*/*z* 85) using HCD type of fragmentation spectra as input. Fragments that yield structural information from the entire carnitine moiety would offer an ideal means to classify fragmented acylcarnitine species as it is common to all members of the class. As can be seen in Figure 2A, a combination of the mass fragment [C_4_H_5_O_2_]^+^ and a neutral loss of C_3_H_9_N (i.e., trimethylamine), covers the entire carnitine molecule, whereas larger carnitine-related fragments, i.e., dehydrated carnitine, are absent or of low abundance in the fragmentation spectrum (Figure 2B). Moreover, the mass fragment [C_4_H_5_O_2_]^+^ and neutral loss C_3_H_9_N are the two most intense features of the spectrum. Further inspection of merged HCD spectra of annotated acylcarnitines in MAGMa showed the fragment [C_4_H_5_O_2_]^+^ and neutral loss C_3_H_9_N to be present in all merged fragmentation spectra, indicating a combination of those two can be used for acylcarnitine classification. The fragment annotation as proposed by MAGMa for acylcarnitines was also compared to literature postulations (Yang et al., 2007; Zuniga and Li, 2011) and found to be consistent.

**Figure 3:**
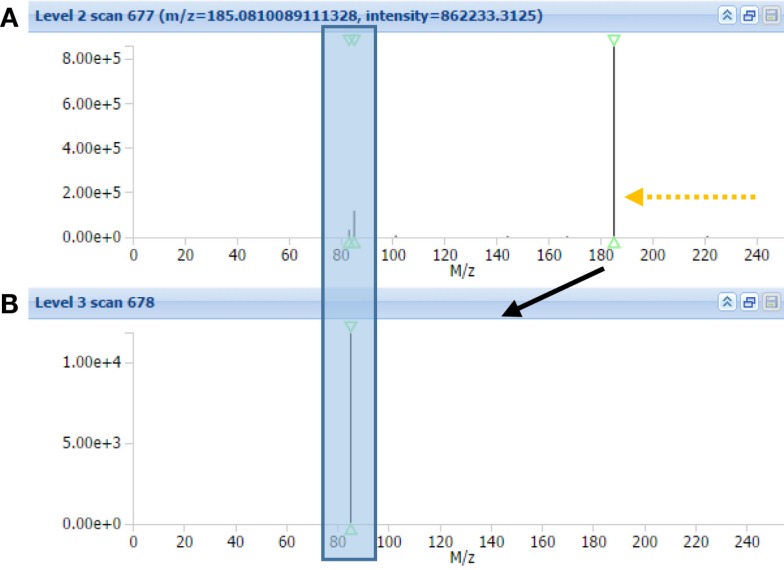
**MAGMa screenshot of CID-MS^2^ spectrum (A) and CID-MS^3^ spectrum (B) of HMDB-annotated tiglylcarnitine**. The MS^3^ scan originates from the highest abundant MS^2^ ion. An arrow indicates the neutral loss of C_3_H_9_N, whereas a box captures the C_4_H_5_O_2_ fragment.

Using CID fragmentation, the mass fragment [C_4_H_5_O_2_]^+^ and the neutral loss C_3_H_9_N both occurs, as can be seen in Figure 3 for one annotated acylcarnitine; however, the mass fragment [C_4_H_5_O_2_]^+^ is obtained with fivefold lower intensities compared to HCD fragmentation, whereas the neutral loss occurs at similar abundance. Moreover, most of the MS3 scans obtained in CID-MS*^n^* did not provide additional fragment information, being empty or repeating the MS2 fragment [C_4_H_5_O_2_]^+^ as in Figure 3B. Thus, based on the comparison of CID-MS*^n^* and HCD-MS2 type of fragmentation for acylcarnitines, the HCD-MS2 fragmentation was found to be the preferred fragmentation type to classify and annotate this class of compounds. Therefore, the remainder of the study is mostly based on HCD fragmentation.

**Figure 4:**
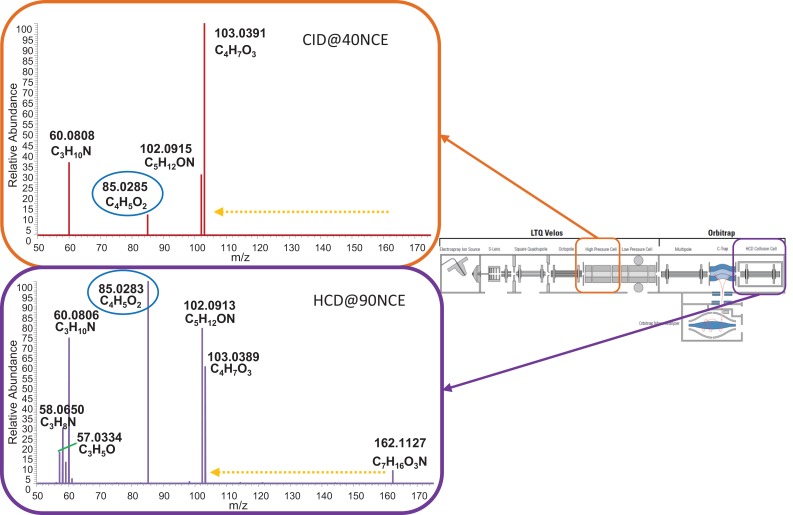
**Direct infusion MS/MS data of l-carnitine in CID (top) and HCD (bottom) mode in the *m*/*z* range 50–175**. Both energies (CID@40NCE and HCD@90NCE) represent collision energies where the key fragment C_4_H_5_O_2_, marked with a blue oval, is an abundant ion in the respective fragmentation spectra. The yellow dashed arrow indicates the neutral loss of C_3_H_9_N. Boxes indicate where the fragmentation took place in a schematic representation of the hybrid mass spectrometer.

Figure 4 shows the CID-MS^2^ and HCD-MS^2^ spectra of l-carnitine obtained by direct infusion of the reference compound (see Sections “Methods” and “NanoMate Direct Infusion Measurements”), confirming the presence of the mass fragment [C_4_H_5_O_2_]^+^ and the neutral loss C_3_H_9_N (resulting in C_4_H_7_O_3_; *m*/*z* 103.0390) upon carnitine fragmentation. As in the LC-MS experiments, the abundance of the key mass fragment is much lower for the displayed CID-MS^2^ fragmentation spectrum, supporting HCD as the preferred fragmentation type to classify and annotate acylcarnitines. Based on the LC-MS and direct infusion experiments, we concluded that in the case of HCD type fragmentation, a mass fragmentation filter of the fragment mass [C_4_H_5_O_2_]^+^ (*m*/*z* 85.0284) and a neutral loss of C_3_H_9_N (*m*/*z* 59.0735) can be used to annotate acylcarnitines; in case of CID-MS*^n^*, the same filter could be applied (valid for acylcarnitines up to 357 *m*/*z* due to the 1/3 cut-off rule as a result of the Ion trap configuration), but it would work less well for lower abundant acylcarnitines.

### HCD-MS^2^ fragmentation spectra: Different collision energies are required to obtain sufficient structural information for classification and further annotation of the acyl moiety

Low fragmentation energies usually result in the loss of the more labile side groups of metabolites, such as a carboxyl or hydroxyl group, whereas higher collision energies tend to break up molecular structures into smaller, energetically stable fragments (Watson and Sparkman, 2007). Therefore, the probability of finding a unique combination of fragments or neutral losses for a specific metabolite class increases if multiple fragmentation energies are used. Moreover, it is likely that more structural information can be obtained from a combination of different collisional energies.

**Figure 5:**
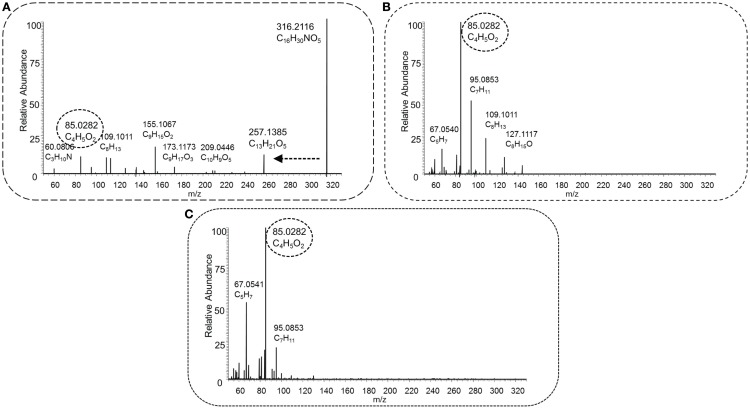
**HCD type MS^2^ fragmentation spectra obtained at low [30 NCE, (A)], middle [70 NCE, (B)], and high [110 NCE, (C)] energy for precursor mass 316.2118 *m*/*z*, with the proposed elemental formula of C_16_H_29_NO_5_**. The key fragment C_4_H_5_O_2_, marked with a dashed oval, and the neutral loss C_3_H_9_N, indicated by a dashed arrow, occur in the spectra, and this fragmented metabolite could be annotated as an acylcarnitine.

**Figure 6:**
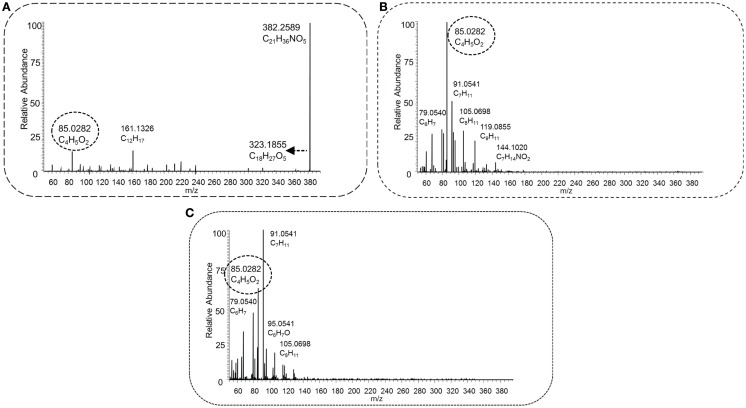
**HCD type MS^2^ fragmentation spectra obtained at low [30 NCE, (A)], middle [70 NCE, (B)], and high [110 NCE, (C)] energy for precursor mass 382.2588 *m*/*z*, with the proposed elemental formula of C_21_H_35_NO_5_**. The key fragment C_4_H_5_O_2_, marked with a dashed oval, and the neutral loss C_3_H_9_N, indicated by a dashed arrow, occur in the spectra, and this fragmented metabolite could be annotated as an acylcarnitine.

Figure 5 shows the low (Figure 5A), middle (Figure 5B), and high (Figure 5C) energy HCD-MS^2^ fragmentation spectra for a novel acylcarnitine structure (i.e., not present in Scifinder) that was detected and annotated in this study. The three spectra are clearly different with the neutral loss from the carnitine substructure present in the low energy spectrum, but not observed at higher energies. In contrast, the key fragment C_4_H_5_O_2_ observed at low abundance in Figure 5A is the base peak in the middle and higher collision energy spectra (Figures 5B,C). The different energies show complementary fragments derived from the acyl part of the acylcarnitine molecule, which assists in further structural characterization. This use of three HCD-MS^2^ fragmentation energies resulted in complementary structural information and enabled detection of both the key neutral loss and fragment that together form the acylcarnitine mass fragmentation filter and the detection of structurally informative acyl-derived fragments (Figures 5 and 6).

### Application of the mass fragment and neutral loss filter to classify fragmented acylcarnitines in pHILIC-MS and HILIC-MS data-dependent fragmentation runs

The initial MAGMa analysis enabled the annotation of the LC-MS/MS files with candidate acylcarnitine structures from HMDB and the design of a mass fragmentation filter to classify acylcarnitines based on the proposed substructure annotations. Subsequently, this filter was applied to the data-dependent (information dependent, untargeted) HCD fragmentation data of the two urine extracts run with pHILIC gradients (urine 1 and 2), and two urine extracts run with a HILIC-MS/MS gradient (urine 3 and 4). This led to the classification of 22 different acylcarnitines including carnitine itself, 10 of which occurred as multiple isomers, based on concurrence of the key neutral loss and the key fragment in the MS^2^ fragmentation spectra of detected and fragmented acylcarnitine species. Dehydrocarnitine does not display the typical fragmentation as observed for carnitine and was therefore not classified. The classified acylcarnitines ranged from *m*/*z* 162.1125 ([M + H]^+^), identified as carnitine, to *m*/*z* 318.1911 ([M + H]^+^), annotated as the conjugate of suberic acid (octane-1,8-dioic acid) and carnitine (MSI MI level 3). Notably, out of the 22 annotated acylcarnitines, 11 were not present in HMDB and were thus previously not annotated with MAGMa. Furthermore, one candidate acylcarnitine structure is present in HMDB for four annotated acylcarnitine isomer pairs.

### Application of mass fragment and neutral loss filter on targeted fragmentation spectra of suspected low abundance acylcarnitine species detected during untargeted metabolomics

Upon studying full scan HILIC-MS data of extracts 5, 6, and 7 of human urines, 27 masses of potential lower abundant acylcarnitine structures not previously fragmented and annotated in urines 1–4 were included in parent ion lists (see HILIC-MS/MS). Based on the resulting HCD fragmentations that could be obtained in sufficient quality for 19 of them, 18 masses were confirmed to be acylcarnitines. Most of these acylcarnitines had higher *m*/*z* values, i.e., >330 Da, than those annotated during the data-dependent fragmentation runs. An exception is the acylcarnitine with elemental formula C_13_H_20_NO_6_ ([M + H]^+^, *m*/*z* 286.1285), which could be annotated as a conjugate of C_6_H_6_O_4_ [possibly 2-furyl(hydroxy)acetic acid or 2,3-methylenesuccinic acid] and carnitine (MSI MI level 3).

### Scifinder analysis of annotated acylcarnitines

Scifinder, a widely used, comprehensive, and well-curated compound database, was used to evaluate the present findings^7^. All annotated acylcarnitine formulae were searched in Scifinder, returning “all hits,” i.e., all Scifinder known structures with that elemental formula. Subsequently, the refine panel in Scifinder was used and a substructure search based on the carnitine structure was performed within all the hits for a given elemental formula, returning “carnitine refined hits.” Finally, if one or more acylcarnitine structures were returned by Scifinder, the number of “references to the top hit” was noted as an indication of how well-known the structure is.

**Figure 7:**
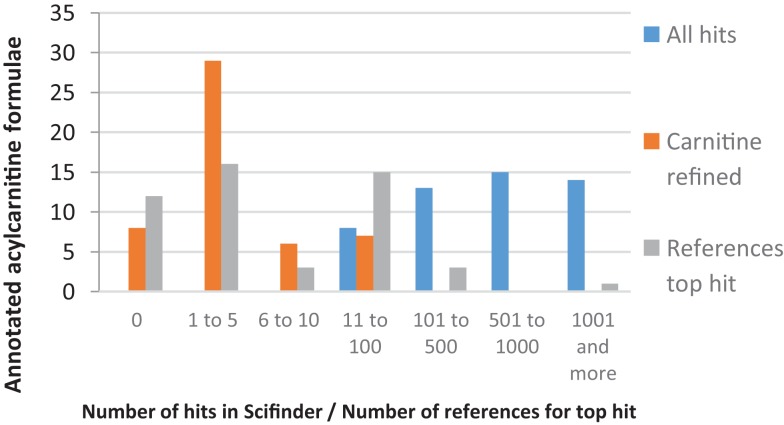
**Combined bar plot of the number of annotated acylcarnitine formulae for: (i) “All hits,” i.e., the number of Scifinder hits for elemental formulas (EF) of the 50 annotated acylcarnitine formulae, (ii) “Carnitine refined hits,” i.e., the number of Scifinder hits with the refinement of a carnitine substructure, and (iii) “References top hit,” i.e., the number of references to the most cited acylcarnitine (top hit)**.

Figure 7 shows histograms of the Scifinder analysis for all 50 annotated acylcarnitines, including those annotated using data-dependent fragmentation (22), those annotated using a parent ion list (18), and the annotated acylcarnitine isomers (10). Detailed figures can be found in Table S1 in Supplementary Material.

Of the 22 acylcarnitines annotated using data from untargeted fragmentation, 12 had more than 10 references for the most cited acylcarnitine structure in Scifinder, indicating that they are relatively well-studied and characterized. However, accurate mass fragmentation spectra of underivatized forms of these acylcarnitines are still sparse, since the acylcarnitines were identified in the references based on either GC-MS or LC-MS data combined with nominal mass fragmentation. Moreover, 7 of the 22 elemental formulae resulted in 2 or fewer references for the most cited acylcarnitine structure in Scifinder. For example, no acylcarnitine structure with elemental formula C_12_H_18_NO_5_ ([M + H]^+^, *m*/*z* 256.1179) was present in Scifinder. The corresponding acyl-moiety with elemental formula C_5_H_4_O_3_ could match four possible structures in HMDB, with 3-furoic acid (HMDB 004444) being a likely candidate based on natural abundance in human urine. However, the HCD spectrum does not indicate the presence of a furan moiety, nor any other fragment to allow confirmation of one of the candidate acyl structures. Of the acylcarnitines annotated using a parent ion list, seven did not yield any hits in Scifinder with their elemental formula and substructure refinement as input (Table S1 in Supplementary Material). This shows that targeted (data independent) fragmentation data in combination with a mass fragmentation filter can be used to structurally classify observed mass peaks in untargeted mass spectrometry experiments.

Taking all 50 annotated acylcarnitines together, half of them have 5 or less references for the most cited acylcarnitine structure, indicating that few studies could reliably annotate or identify these acylcarnitines. This Scifinder analysis shows that the presented workflow yields new knowledge from untargeted metabolomics experiments by generating data-dependent accurate mass fragmentation data and providing robust classification of acylcarnitine species both present and absent in HMDB.

### Structural annotation of the acyl moiety

After classification of an acylcarnitine (MSI MI level 3), more information on the fragmented metabolites can be obtained by comparison of the obtained fragmentation spectra with database spectra (if present, MSI MI level 2) or studying the fragments derived from the acyl part. The fragmentation data was analyzed as described in Section “Metabolite Annotation.” This resulted in three MSI level 2 annotations for acylcarnitines and their fragmentation data present in MzCloud or Metlin. In addition, dl-carnitine could be annotated with MSI MI level 1, since the fragmentation spectrum of the urinary compound matched with that of an authentic standard. Accurate mass fragmentation data for the remaining 46 acylcarnitines could not be found; therefore, acyl-derived mass fragments and neutral losses (from the suspected acyl-parent ion) were studied. For example, double CH_2_O_2_ and/or H_2_O loss appeared to be indicative for a di-carboxylated acyl moiety, like suberic acid and dodecanedioic acid. To explore another route to structural annotation of the acyl moiety, acyl-derived fragments were manually uploaded into MAGMa to find candidate acyl structures. All annotations of the studied acylcarnitines can be found in Table S1 in Supplementary Material.

Three cases are described here in more detail. As described in Section “Application of the Mass Fragment and Neutral Loss Filter to Classify Fragmented Acylcarnitines in pHILIC-MS and HILIC-MS Data-Dependent Fragmentation Runs,” Figure 5 shows the fragmentation spectra of an acylcarnitine with elemental formula C_16_H_30_NO_6_ ([M + H]^+^, *m*/*z* 316.2118). Its acyl part is represented by several fragments at lower fragmentation energy, the largest being C_9_H_17_O_3_. Neutral losses of H_2_O and CH_2_O_2_ from this fragment indicate the presence of a carboxyl group within the acyl moiety. In order to obtain candidate structures for the acyl moiety based on the observed fragments, a list of acyl-derived fragment masses and the suspected “parent mass” was uploaded into MAGMa, and HMDB and Pubchem were queried using default MAGMa parameters. The nine resulting HMDB candidates all had the elemental formula of C_9_H_16_O_3_ but none had a free carboxyl group. Pubchem resulted in 2,158 candidates (all C_9_H_16_O_3_), with four hydroxylated C9:1-fatty acids among the top 35 metabolites (based on candidate scores). Thus, the C_16_H_30_NO_6_ ([M + H]^+^ acylcarnitine could be annotated as a C9:1-OH-acylcarntine (MSI MI level 3).

Figure 6 shows the spectra of the annotated acylcarnitine with elemental formula C_21_H_36_NO_5_ ([M + H]^+^, *m*/*z* 382.2588), which represents a C_14_H_22_O_3_-carnitine conjugate. The mass fragments and losses present at lower and higher energies, i.e., the combined loss of C_2_H_6_O_3_ and the mass fragments C_12_H_17_ and C_9_H_11_ {[Molecular Fragment (MF)]^+^}, revealed no indicative losses of a carboxyl group. The acyl moiety likely consists of a branched, unsaturated alkyl chain, since the ring double bond equivalent of C_14_H_22_O_3_ is 4. The acyl-derived fragment masses and suspected parent mass were queried, and HMDB returned one metabolite, geranyl acetoacetate, with the correct elemental formula; however, two fragments, C_13_H_19_ and C_12_H_17_ ([MF]^+^), could not be explained by MAGMa based on this structure, and many others had a high penalty score (≥5). Pubchem, however, returned 1,743 candidate structures with the elemental formula C_14_H_22_O_3_, of which a 3-hydroxytetradeca-5,8,11-trienoic acid, was listed in the top 5 (based on candidate scores), and appears to be a plausible candidate. Therefore, this acylcarnitine could be annotated as a C14:3-OH-acylcarnitine (MSI MI level 3). Interestingly, several of the observed fragments in Figures 5A,B (i.e., C_7_H_7_, *m*/*z* 91.0542; C_7_H_9_, *m*/*z* 93.0699; and C_7_H_11_, *m*/*z* 95.0855, all [MF]^+^) were also found in other high mass acylcarnitines (i.e., >330 *m*/*z*) at higher collision energies, indicating similar substructures in the acyl moiety of these metabolites.

**Figure 8:**
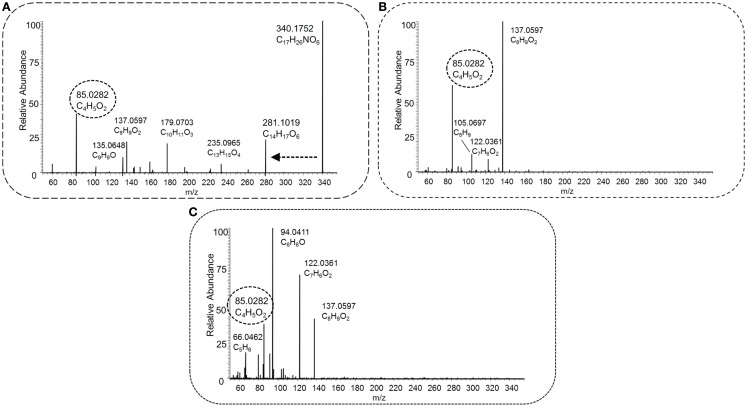
**HCD type MS^2^ fragmentation spectra obtained at low [30 NCE, (A)], middle [70 NCE, (B)], and high [110 NCE, (C)] energy for precursor mass 340.1755 *m*/*z*, with the proposed elemental formula of C_17_H_25_NO_6_**. The key fragment C_4_H_5_O_2_, marked with a dashed oval, and the neutral loss C_3_H_9_N, indicated by a dashed arrow, occur in the spectra, and this fragmented metabolite could be annotated as an acylcarnitine.

Figure 8 shows the fragmentation spectra of a novel detected acylcarnitine (i.e., not present in Scifinder) with the elemental formula C_17_H_26_NO_6_ ([M + H]^+^ and *m*/*z* 340.1755), which shows mass fragments different from most other acylcarnitine spectra observed in this study. Its conjugated acyl moiety has the elemental formula of C_10_H_12_O_4_ (which implies five ring double bond equivalents), likely to be caused by the presence of an aromatic ring. Further evidence is provided by the fragments C_6_H_7_ and C_5_H_6_ [MF]^+^. A (radical) loss of CH_3_ was also observed, indicating a methoxy substitution on the aromatic ring. After collecting the acyl-derived fragment masses and suspected parent mass, HMDB returned 13 candidates with the elemental formula C_10_H_12_O_4_, of which 2-hydroxy-3-(4-methoxyphenyl)propanoic acid is a potential candidate. However, the fragment C_7_H_6_O_2_ (*m*/*z* 122.0361 [MF]^+^) does not fit well within the structure without breaking the aromatic ring. Pubchem yielded 1,520 candidates based on the input mass fragments, all with elemental formula C_10_H_12_O_4_. Within the top 20 hits (based on candidate scores), dihydroferulic acid and 5 structurally related isomers were present candidate structures for the acyl moiety. The fragment C_7_H_6_O_2_ [MF]^+^ can be explained with an intact aromatic ring, indicating that the hydroxyl group is substituted to the aromatic ring. Altogether, this acylcarnitine could be annotated as conjugate of carnitine and C_10_H_12_O_4_, with a likely candidate being 3-(4-hydroxy-3-methoxyphenyl)propionic acid (i.e., dihydroferulic acid) or a structurally related isomer (MSI MI level 3).

## Discussion

This study explored the use of accurate mass fragmentation approaches in untargeted and targeted HILIC-MS metabolomics experiments to obtain increased confidence in metabolite annotations. Human urine extracts, representing a complex mixture of metabolites offered a good test case. Acylcarnitines, metabolites involved in energy metabolism (Frayn, 2010) are relatively abundant in urine and identified as biomarkers for various related diseases (Adams et al., 2009; Patterson et al., 2009; Luan et al., 2014). Initial metabolite annotation of candidate metabolites present in HMDB resulted in the matching of 12 candidate acylcarnitines to fragmentation data files using the MAGMa interface (Ridder et al., 2014b). These annotations then allowed us to define a mass fragment and neutral loss filter to classify detected and fragmented acylcarnitines from standard pHILIC and HILIC LC-MS runs of urine extracts. With the use of this filter in the context of an untargeted metabolomics experiment, substantially more acylcarnitines could be reliably annotated from each run, yielding a total of 50 uniquely classified acylcarnitine species (including multiple observed isomers) in both untargeted (32) and targeted (18) fragmentation runs studied (MSI MI level 3). Using our metabolomics platform, these confident annotations were previously not possible; however, using the methodology described in this study, we could enhance the annotation power of our platform for acylcarnitines, and at the same time collect novel accurate mass fragmentation data for this set of acylcarnitines.

Previous work showed the value of parent and neutral loss monitoring in a quadrupole ion trap for targeted screening of acylcarnitines in biological samples (McClellan et al., 2002; Shigematsu et al., 2002; Paglia et al., 2008; Rinaldo et al., 2008). In addition, several studies applied multiple reaction monitoring (MRM) type of approaches using the 85 *m*/*z* nominal mass fragment or the neutral loss of 60 to target specifically for acylcarnitines (Maeda et al., 2008; Kivilompolo et al., 2013; Peng et al., 2013b) or specific derivatization to probe for acylcarnitines (Minkler et al., 2005). It should be noted that all the above mentioned targeted approaches required dedicated sample preparation, used nominal mass spectrometers, and in many cases applied derivatization to enhance the sensitivity of the method. Our study showed that the use of such parent and neutral loss monitoring within the context of untargeted high-resolution metabolomics experiments is very valuable in enabling robust annotations for a biologically relevant class of metabolites without the use of specific sample extractions, chromatographic gradients, or complex MRM methods.

**Figure 9:**
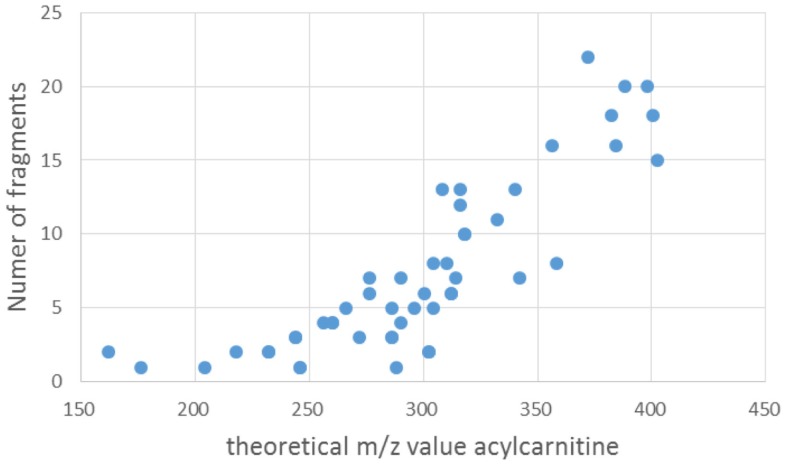
**Scatterplot of the number of fragments counted between *m*/*z* 85 and the mass value of the precursor ion (i.e., fragmented annotated acylcarnitine) vs. the theoretical mass value of the acylcarnitine in positive ionization mode**.

Zuniga and Li (2011) reported the most comprehensive study to date using a similar, but nominal, mass filter for acylcarnitine detection, and reported 355 acylcarnitine species (non-derivatized) in a 2-h UPLC gradient. Unfortunately, these are not yet included in the HMDB database, and 16 out of the 355 substances reported in the study were added to Scifinder. A possible reason could be that no definite elemental formulas (EF) were assigned to all reported acylcarnitines, because the annotations were made on the basis of nominal mass spectra and postulated fragment structures. In contrast, our approach could provide more confident annotations based on the accurate mass full scan and MS^2^ fragmentation spectra, allowing confident elemental formula assignments and classification as acylcarnitines, as was very recently underlined by Sumner et al. (2014). Comparison of our data to the spectra obtained by Zuniga and Li was made, and for the three presented cases in Figures 5, 6, and 8, the most likely corresponding acylcarnitines, as monitored and labeled with their nominal observed mass and isomer letter in their supporting information by Zuniga and Li, are 316[E] (fragment 127.1), 382[C] (fragments 161.3, 119.2, 95.1, and 91.0), and 340[A + B] (fragments 179.2 and 137.0) for annotated acylcarnitines in Figures 5, 6, and 8, respectively (with corresponding fragments to our study between brackets). It should be noted that the 2-h UPLC gradient allowed for separation of structurally related acylcarnitines that were not separated in our 15 (pHILIC) and 30 (HILIC) minute gradients. All annotated masses in this study could potentially be matched with nominal masses found in Zuniga and Li’s extensive study, but a detailed comparison for all annotated acylcarnitines is hampered by differences in chromatography and mass spectrometry methodology used. An advantage of our approach is the use of multiple fragmentation energies resulting in both higher-mass fragments (at low collision energy) and lower-mass fragments (at high collision energy), creating a unique fingerprint and enabling further structural characterization of the acyl moiety than in previous studies. The combination of multiple energies also allowed more fragments to be detected; and the number of fragments increased with increasing molecular mass (Figure 9). The number of fragments has a great impact on the structural information that can be gathered from a fragmentation spectrum. It should be added that annotated isomeric acylcarnitines generated similar fragmentation spectra (see also Section “Limitations of the Current Study”). Remarkably, 27 unique acylcarnitine EF returned no hits upon querying in HMDB; indicating the need for improving database coverage to facilitate metabolite annotations (see Compound and Spectral Databases for Metabolite Annotation). Therefore, alternative ways to characterize the structure of the acyl moiety were explored in this study. We compared fragmentation spectra to spectral databases and by studying neutral losses and using MAGMa to find candidate metabolites (see Scifinder Analysis of Annotated Acylcarnitines), thereby revealing the unexpected acyl moiety dihydroferulic acid or a structurally related isomer for one of the annotated acylcarnitines (Table S1 in Supplementary Material).

### Advantages of using MAGMa for initial annotation of urine extracts with HDMB candidates

- MAGMa annotation of the LC-MS fragmentation data with candidates from an appropriate database [in this study HMDB (Wishart et al., 2013)], provided a quick overview of a diverse range of candidate metabolites present in the urine extracts.
- The fragment annotations proposed by MAGMa helped to quickly recognize specific fragmentations of acylcarnitines.

### Advantages of using an accurate mass fragmentation filter to classify compounds within the context of untargeted metabolomics experiments

- Measurement of fragmentation data in untargeted metabolite profiling experiments allows MSI MI level 3, and sometimes 2, annotations where otherwise only level 4 would be possible. A similar trend was previously observed for CID-MS*^n^* approaches applied to plant secondary metabolites (Van Der Hooft et al., 2012b).
- Accurate fragment mass values allow more reliable elemental formula assignments of the fragment ions and molecular ions, resulting in more reliable metabolite annotations (Sumner et al., 2014).
- Acylcarnitines were reliably annotated in urine datasets that also contain fragmentation data of many other metabolite classes like amino acids and purines. This allows different classes of metabolites to be studied in the same datasets using the same approach.

### Limitations of the current study

- Sample preparation and chromatography used were generic and not optimized for acylcarnitine detection, resulting in a lesser chromatographic resolution for acylcarnitines than obtained in some other others targeting acylcarnitines, e.g., Zuniga and Li (2011) and Gucciardi et al. (2012).
- The data-dependent fragmentation approach in combination with the mass fragmentation filter did not result in annotation of all studied acylcarnitines, as for 18 lower abundant acylcarnitines a targeted fragmentation approach was needed to obtain fragmentation spectra enabling their classification as acylcarnitines.
- Chromatographically separated, isomeric acylcarnitines (sharing the same elemental formula) could not be discriminated based on their fragmentation patterns and need additional spectral information (e.g., NMR spectroscopy) to confidently discriminate them. This phenomenon is commonly observed in mass spectrometry data, especially for stereoisomers, with some exceptions to this rule (Van Der Hooft et al., 2011).

### Compound and spectral databases for metabolite annotation

Our metabolite annotation would benefit from an increased coverage in compound databases (like HMDB) and spectral databases (like mzCloud, Metlin, and MassBank). The availability of more fragmentation spectra of reference compounds would facilitate the design of more mass fragmentation filters such as the one described in our study. Despite the fact that the MzCloud database (see text footnote 5) and Massbank^8^ provide fragmentation data for many reference compounds, often in both ionization modes, at different energies, and from different instruments, MzCloud and Massbank contain spectral data for only 5 and 7 acylcarnitine structures, respectively, which is a small number compared to the 50 annotated in our study. In fact, in our study, only 3 of the reported acylcarnitines could be matched to database fragmentation spectra searched for in different spectral databases, “upgrading” the level 3 annotation to level 2, apart from carnitine, for which we could obtain in-house reference data allowing for a MSI MI level 1 identification. We also applied LipidSearch (ThermoScientific software) to our fragmentation data, but the software did not return any acylcarnitine candidates matched to the fragmentation data. Furthermore, as mentioned before, HMDB does not cover the majority of acylcarnitine EF annotated in this study. Emerging metabolite annotation software tools like MAGMa will benefit from an increased coverage of compound databases such as HMDB since they serve as input for candidate metabolites. Finally, standardization of metabolomics data reporting, as promoted by COSMOS^9^ and MetaboLights and the MSI initiative (Sumner et al., 2007, 2014; Salek et al., 2013a,b) will allow metabolomics researchers to (i) build on each other’s findings in method development and data analysis by easier exchange of data and protocols, and (ii) facilitate the search for earlier reported annotated metabolites and their spectral data, thereby facilitating metabolite annotations of present and future studies.

### Future research directions

Implementation of mass fragmentation approaches into routine untargeted high-resolution metabolomics experiments would benefit from: (i) finding a working compromise for the coverage of uniquely fragmented metabolites and the need for multiple energies or fragmentation depths (and thus scan cycle times); and (ii) creating more mass fragmentation filters to classify metabolite features and support robust metabolite annotation, thereby reducing the number of MSI MI level 4 annotations in untargeted metabolomics experiments:

(i) Data-dependent fragmentation is a stochastic process, resulting in mass peaks to be fragmented in one run and not in another. The use of different collision energies for HCD fragmentation proved to be essential to get as much structural information as possible on the annotated acylcarnitines (see HCD-MS^2^ Fragmentation Spectra: Different Collision Energies are Required to Obtain Sufficient Structural Information for Classification and Further Annotation of the Acyl Moiety), and is important in untargeted mass spectrometry to get structural information on diverse set of compounds present in biological extracts (Madala et al., 2012). There is a compromise between the number of compounds for which fragmentation data can be acquired and the amount of fragment data generated per compound. Very recently, several ways to improve coverage of fragmented masses during data-dependent analysis have been postulated, i.e., by using gas-phase fractionation (Calderón-Santiago et al., 2014), by so-called SWATH analysis (Roemmelt et al., 2014) through “delayed fragmentation” as was proposed for peptide fragmentation in proteomics (Savitski et al., 2011), or by a combination of data dependent and independent fragmentation approaches (Hoffmann et al., 2014). It should be noted that some of those fragmentation strategies require sophisticated tools to analyze the resulting data sets as a result of multiple precursor ions being simultaneously fragmented. Therefore, extension of this work will be primarily focused on finding the optimal compromise between metabolite coverage and structural information using narrow isolation windows.
(ii) The present findings showed that metabolite classification (i.e., fatty acids, imidazole-containing, carnitine-related, etc.) based on key mass fragments and neutral losses is a promising approach within the context of untargeted mass spectrometry. Moreover, the approach not only enables more complete annotations within complex metabolomics datasets but also reduces the number of candidate metabolites to be considered for a detected mass feature, e.g., based on database queries on elemental formula, from more than 100 to only a handful (see Figure 7 and Table S1 in Supplementary Material). Organic molecules consist of recurring subunits, often decorated with different side groups and chains. Therefore, future work will aim to derive more relevant structural key mass fragments and neutral losses for other classes of compounds present in complex biological samples, like human urine, by integrating expert knowledge and automated approaches.

## Conclusion

Metabolite classification based on a specific set of observed fragments and neutral losses proved to be a successful approach in enabling robust annotations of mass peaks observed in untargeted mass spectrometry. MAGMa can successfully annotate acylcarnitine structures present in HMDB to fragmented acylcarnitine masses in complex biological samples. Based on the acylcarnitine CID and HCD fragment and neutral loss annotation, a selective mass fragmentation filter was constructed. Application of that filter to HCD fragmentation data obtained using data dependent and targeted fragmentation methods led to the annotation of 50 urinary acylcarnitines of which most had not been reliably annotated before using a high-resolution HILIC-MS approach. The annotation approach we describe shows that within the context of untargeted high-resolution mass spectrometry based metabolomics experiments, reliable metabolite annotations can be achieved using standard, high-throughput untargeted approaches in combination with mass fragmentation filters that allow for metabolite classifications.

## Author Contributions

JvdH designed the research, conducted the experiments, analyzed the results, and wrote the manuscript. LR, MPB, and KB contributed to helpful discussions on the results and writing of the manuscript.

## Conflict of Interest Statement

The authors declare that the research was conducted in the absence of any commercial or financial relationships that could be construed as a potential conflict of interest.

## Supplementary Material

The Supplementary Material for this article can be found online at http://www.frontiersin.org/Journal/10.3389/fbioe.2015.00026/abstract. All mzXML files of human urine extracts 1–7 that associated with this study are publicly available from the “AcylcarnitineManuscript” folder of the “JVanDerHooft_Public” repository at the MetabolomeExpress (Carroll et al., 2010) website (https://www.metabolome-express.org).

Table S1 **Overview of acylcarnitines including spectral properties and Scifinder analysis**. The rows represent the annotated acylcarnitine metabolites (including carnitine), and the rows (left to right) represent: the human urine extract number for which the scan number and retention time are noted in the Table (Urine file fragm); the scan number of the full scan in which the acylcarnitine is precursor ion (# MS1); the retention time in minutes (RT); the theoretical mass of the ionized acylcarnitine (*m*/*z* [M + H]^+^); the elemental formula of the ionized acylcarnitine (EF [M + H]^+^); the number of hits Scifinder returns on querying the (neutralized) elemental formula of the acylcarnitine (Hits Scifinder); the number of hits in Scifinder remainin after refining based on the (decharged) substructure of carnitine (Refine: Carnitine); the number of references to the most cited acylcarnitine returned by Scifinder (# refs Top hit); the number of fragments counted between *m*/*z* 85 and the precursor ion (# fragm 85 – precurs); the number of oxygen atoms in the acylcarnitine minus the three carnitine oxygen atoms (# O atom minus 3); the carbon to hydrogen ratio (C:H), the presence (Y) or absence (N) of the annotated acylcarnitines in HMDB (Present in HMDB?); the annotation/identification of reported acylcarnitines, using the nomenclature of Zuniga and Li (2011) for acylcarnitines not present in databases (Annotation/Identification); the Metabolomics Standard Initiative Metabolite Identification level of the reported acylcarnitines (MSI MI level); information on the metabolite annotation process (Spectra compared to/fragment analysis); and the SMILES string for level 2 or 1 identified structures (SMILES).

## Footnotes

^2^www.hmdb.ca

^3^http://www.massmatrix.net/mm-cgi/downloads.py

^4^http://metlin.scripps.edu/

^5^http://www.mzcloud.org

^6^http://www.hmdb.ca/

^7^https://scifinder.cas.org/scifinder

^8^http://www.massbank.jp/

^9^http://cosmos-fp7.eu/
